# Multi-Class Skin Lesions Classification Using Deep Features

**DOI:** 10.3390/s22218311

**Published:** 2022-10-29

**Authors:** Muhammad Usama, M. Asif Naeem, Farhaan Mirza

**Affiliations:** 1 School of Computing, National University of Computer & Emerging Sciences, Islamabad 44000, Pakistan; 2 School of Engineering, Computer and Mathematical Sciences, Auckland University of Technology, Auckland 1010, New Zealand

**Keywords:** skin cancer, augmentation, deep learning, moth flame optimization, SVM, feature optimization, transfer learning, deep features

## Abstract

Skin cancer classification is a complex and time-consuming task. Existing approaches use segmentation to improve accuracy and efficiency, but due to different sizes and shapes of lesions, segmentation is not a suitable approach. In this research study, we proposed an improved automated system based on hybrid and optimal feature selections. Firstly, we balanced our dataset by applying three different transformation techniques, which include brightness, sharpening, and contrast enhancement. Secondly, we retrained two CNNs, Darknet53 and Inception V3, using transfer learning. Thirdly, the retrained models were used to extract deep features from the dataset. Lastly, optimal features were selected using moth flame optimization (MFO) to overcome the curse of dimensionality. This helped us in improving accuracy and efficiency of our model. We achieved 95.9%, 95.0%, and 95.8% on cubic SVM, quadratic SVM, and ensemble subspace discriminants, respectively. We compared our technique with state-of-the-art approach.

## 1. Introduction

Skin cancer is one of the worst types of cancer. The two most common types of cancer are non-melanoma and melanoma. Melanoma lesions have a higher mortality rate. However, if the condition is diagnosed early enough, doctors can cure 90% of people. The manual classification of skin lesions is difficult and imprecise due to the considerable similarities between different types of lesions, which leads to incorrect detection. As a result, the automatic classification and detection of lesions utilising image-processing techniques, deep learning, and artificial intelligence can aid in accurately detecting the type of lesion [1].

In 2017, there were 3590 fatalities from 95,360 cases in the United States. Melanoma accounted for 87,110 of these cases. In 2018, there were 13,460 recorded fatalities out of a total of 99,550 cases. In 2018, there were 91,270 melanoma cases. In the United States alone, 104,350 instances of skin cancer were documented in 2019. Men were found to have 62,320 instances while women had 42,030. Melanoma accounted for 96,480 of all skin cancer cases recorded in 2019 (57,220 in males and 39,260 in women). Melanoma claimed the lives of 7320 people in 2019. Every year, more than 15,000 people in the United States die as a result of skin cancer lesions [2,3]. The death rate from melanoma infections may continue to rise in the future.

Skin cancer detection is challenging due to variances in skin textures and injuries. As a result, dermatologists use dermoscopy, a noninvasive procedure, to detect skin abnormalities at an early stage [4]. The gel is applied to the diseased region as the initial step of dermoscopy. The image is then amplified using a magnifying tool [5]. This enlarged picture allows a better analysis of the structure of the lesion region. The detection accuracy is determined by the dermatologist’s experience. According to one study, a dermatologist’s detection accuracy might range between 75% and 84% [6]. A manual diagnosis of skin lesions via dermoscopy, on the other hand, is a time-consuming operation that, even for skilled dermatologists, has a substantial chance of error. As a result, researchers developed a variety of computer-aided diagnostic (CAD) procedures based on machine learning and deep CNN features [7].

Dermatologists can employ CAD systems to rapidly and accurately diagnose skin lesions [8]. The primary phases in a CAD system are the collection of skin image datasets, feature extraction and selection, and classification [9]. When compared to traditional feature extraction approaches, deep feature extraction for skin lesion detection and classification has proven to be extremely important in recent years. Deep features are derived from the fully linked layers of a CNN model, which is then used for classification [10]. Unlike traditional approaches, such as texture, colour, and shape, deep features encompass both local and global information about a picture. The convolutional layer extracts local information from an image, whereas the 1D layers gather global information (global average pooling and fully connected) [11]. Traditional approaches extract shape information, such as HOG, colour, and texture (LBP) independently.

Traditional clinical procedures for diagnosing melanoma are ineffective. Even a skilled dermatologist might make a mistake in accurately diagnosing melanoma. As a result, there is a need for computerised diagnostic systems such as computer-aided detection (CAD) systems or digital dermoscopy. Dermoscopy is the practise of assessing and examining pigmented lesions. It was discovered that dermoscopy can boost the detection rate by 10% to 27%. It is a non-invasive technology for evaluating high-resolution dermoscopic pictures captured by a colour video camera attached to a computer [12]. Based on established algorithms, image processing is used in CAD diagnostic systems that extract features. Following that, the system employs these features to determine whether or not a person has cancer.

### Research Contributions

This paper provides the following contributions:

- **Feature extraction:** feature extracted via transfer learning using two different models Darknet53 and Inception V3;
- **Improving accuracy:** improving classification accuracies using hybrid features extracted from the above two models;
- **Improving efficiency:** improving efficiency via feature reduction using the moth flame optimization algorithm;
- **Performance evaluation:** performance evaluated based on accuracy and efficiency.

The flow of this article is as follows: Section 1 introduces the problem domain. In Section 2, we present past research relevant to our work. The steps involved in experimentation are outlined in Section 3, from dataset balancing to classification. In Section 4, results of experiments are presented, and in Section 5, we discuss the findings; finally, Section 6 summarises the conclusion of our work.

## 2. Related Work

There are many approaches for segmenting and classifying skin lesions in the literature, which use either conventional or deep methodologies. Khan et al. [13] presented a unique approach for skin lesion identification and classification based on probabilistic distribution and feature selection. To partition the lesion region, normal and uniform distributions are used. The features are then taken from the segmented pictures, which are then merged using a parallel fusion approach. The entropy-based method is integrated with the Bhattacharyya distance and variance formulation for feature selection. Publicly available datasets, including ISBI 2016, 2017, ISIC 2018, and PH2, are used to evaluate the suggested method. It achieves accuracy rates of 93.2%, 97.75%, and 97.55%.

Manual skin cancer diagnoses are time-consuming and costly; hence, developing automated diagnostic techniques capable of accurately identifying multiclass skin illnesses is crucial. Khan et al. [14] suggested an automated multiclass skin lesion segmentation and classification method based on deep characteristics. An initial color-controlled histogram intensification of the input images is used (LCcHIV). Then, saliency is determined using a ten-layer proprietary CNN. The resulting heat map is transformed into a binary image. It is then utilized for feature extraction from the segmented colour lesion images. To circumvent the dimensionality curse, an improved moth flame optimization (IMFO) method was developed. The obtained features are merged with an MMCA classifier and KELM is used for classification. Using the ISBI 2016 and 2017, ISIC 2018, and PH2 datasets, the proposed technique achieves 95.38%, 95.79%, 92.69%, and 98.70% accuracy, respectively. The accuracy achieved on the HAM10000 dataset (HAM1000 contains pigmented lesions images for seven different types of lesions) was 90.67%.

Recent work in AI for radiology and radiotherapy relied on algorithms based on deep learning. The performance of the deep learning models may be much superior to conventional machine learning techniques, but for that purpose, larger training datasets are required. In order to overcome this problem, data augmentation has become a common way of increasing training datasets, especially in sectors where large datasets are normally unavailable. Data augmentation seeks to produce new data that are utilised to retrain the model, which shows an increase in performance when tested on different datatsets [15]. To assist in understanding the types of data augmentation approaches used in state-of-the-art deep learning models, a systematic analysis of the literature where data augmentation was performed was used to train a deep learning model using medical images (restricted to CT and MRI). Articles were classified as fundamental, deformable, deep learning, or other data enhancement approaches. In this particular study, the authors aims to provide insight into these methodologies as well as confidence in the models’ validity.

The prediction of skin lesions is difficult even for experienced dermatologists because of the contrast between lesions and surrounding skin. In 2020, an automated computer-aided system was proposed that can help clinicians in detecting different types of lesions in the early stage. Deep learning dilated CNNs are known to increase accuracy with the same computational complexities compared to CNN. To implement this, Ratul et al. [16] chose VGG16, VGG19, MobileNet, and InceptionV3 on the HAM10000 dataset. InceptionV3 showed higher overall accuracy per class; this indicates that inceptionV3 can help in increasing the accuracy for the correct classification of different type of lesions.

Mirjalili [17] developed a unique nature-inspired optimization paradigm termed the Moth-Flame Optimization (MFO) method, which we have also used in our approach for reducing features. The major source of inspiration for this optimizer is the transverse orientation navigation approach used by moths in nature. Moths fly at night by keeping a steady angle with respect to the moon, which is a highly efficient method for travelling large distances in a straight line. These beautiful insects, however, are locked in a useless/deadly spiral journey around artificial lights. This study mathematically models this behaviour and optimises it. On 29 benchmark and seven actual engineering issues, the MFO method is compared against other well- known nature-inspired algorithms. The statistical findings on the benchmark functions demonstrate that this method may provide extremely promising and competitive out- comes. Furthermore, the outcomes of real-world issues indicate the algorithm’s utility in tackling difficult problems with confined and unknown search areas. The research also explores the suggested algorithm’s application in the area of maritime propeller design in order to further evaluate its usefulness in practise.

As a result of our literature review, we realise that a variety of approaches have been discussed in the past with the intention of improving classification accuracies. These approaches include the following: feature extraction, hybrid features, ensembles, and feature optimization. We observed that techniques that were based on the augmentation of data and the extraction of features resulted in a higher accuracy of classification when compared to other methods that were proposed. In light of these revelations, we proposed a strategy that is centred on the augmentation of data and the extraction of features from two CNN models, both of which will be retrained via transfer learning. Another objective of our research is efficiency. So, we will also implement moth flame optimization in order to reduce the number of features and improve our efficiency.

## 3. Proposed Architecture

**Figure 1:**
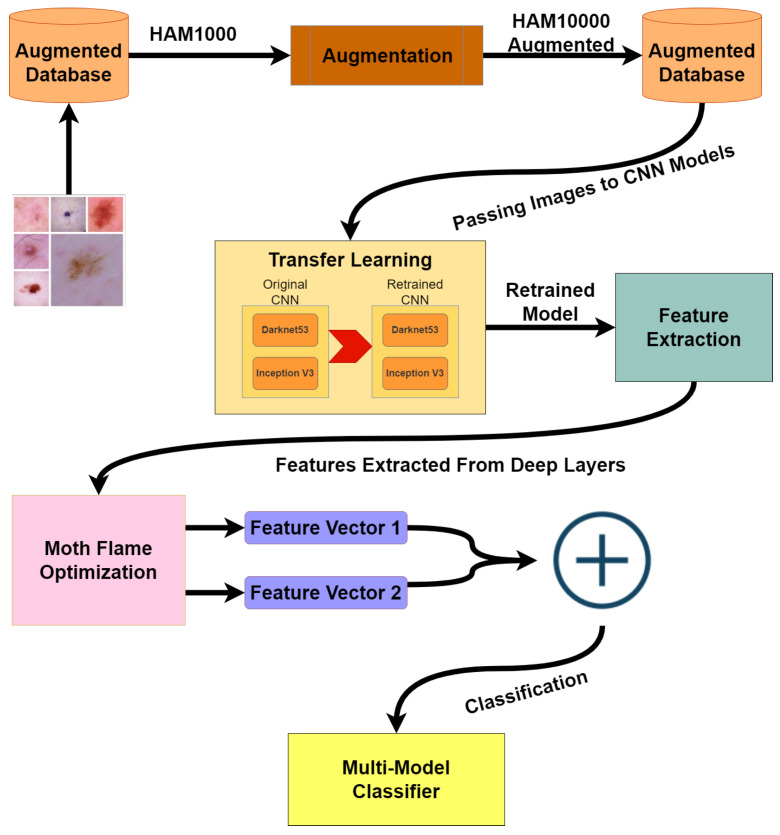
Architecture Diagram.

The architecture diagram in Figure 1 presents our methodology, dataset HAM10000 is highly imbalanced, so the first step is to balance the dataset using augmentation techniques. After the dataset is balanced, the augmented dataset is passed on to two different CNN models: Darknet53 and Inception V3. Both of these models are retrained via transfer learning. In the third step, training and test features are extracted from the dataset by applying activations to the deeper layers of both models. Furthermore, extracted features are reduced by implementing the moth flame optimization algorithm. In the last step, test features are used for classification using cubic SVM; quadratic SVM; linear SVM; linear discriminant; fine, medium, and coarse KNN; ensemble subspace discriminant; and subspace KNN.

### 3.1. Dataset

**Figure 2:**
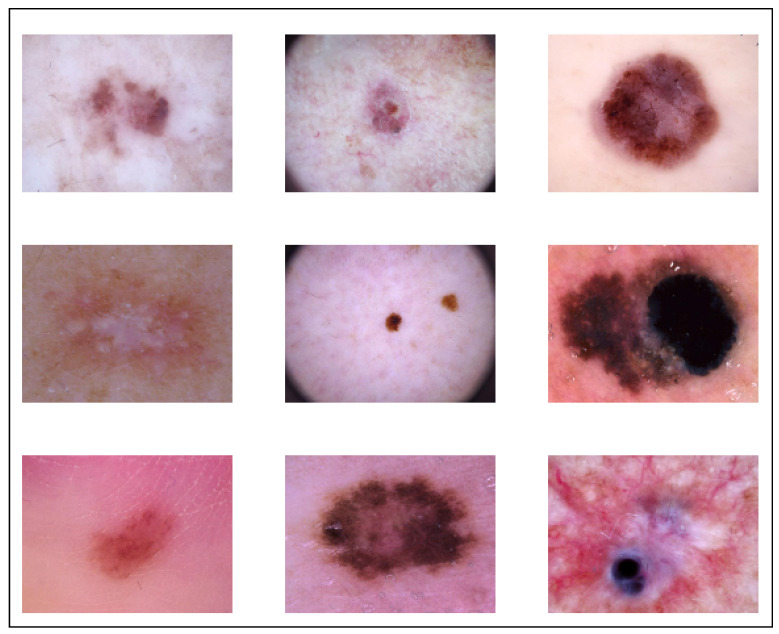
Augmented Dataset.

For the purposes of both training and testing, we used the publicly available dataset known as HAM10000 in our study, which has a total of 10,015 images of dermoscopic pictures that were captured and archived using several modalities. These images include a variety of demographics. The completed dataset includes 10,015 dermatoscopic pictures that are suitable for use as a training set for academic machine learning applications. Cases include a representative collection of all important diagnostic categories in the realm of pigmented lesions, akiec, bcc, bkl, df, mel, nv, and vasc, and the distribution before augmentation was 327, 514, 1099, 115, 1113, 6705, and 142, respectively. After augmentation, the distribution of classes was 981, 1028, 1099, 920, 1113, 6705, and 1136, respectively, which totaled 12,981. The size of original dataset is 2.9 Gigabytes, and it is publicly available. We used raw images shown in Figure 2; it was very difficult to segment areas from images because the shape and size of the effect area varied, and there was a high chance that it may have resulted in cropping important regions.

### 3.2. Data Augmentation

Data augmentation refers to the procedures used to expand the total quantity of data by adding significantly changed copies of previously collected data or freshly produced synthetic data based on previously collected data. When training a machine learning model, it helps prevent overfitting by acting as a regularizer and reducing overfitting. In our approach, we used three different types of augmentation techniques, i.e., contrast enhancement, brightness, and sharpness. We applied these transformations to balance classes that have less images.

1. First, we used contrast enhancement on the dataset. Improvements in contrast increase the visibility of lesions in images by increasing the contrast between objects and their backgrounds.
2. In the second step of augmentation, we increased and decreased the brightness of image to help the model train on images with bright and dark tones.
3. The last augmentation technique we used was to enhance images by increasing and decreasing the sharpness of images.

### 3.3. Transfer Learning

Inception-v3 and Darknet53 are CNNs. We utilised these pretrained networks in our research. These models are pre-trained on the ImageNet dataset. The pretrained network is able to divide photographed objects into one thousand distinct categories. As a direct consequence of this, the network acquired the ability to learn rich feature representations for a diverse set of picture types. The maximum size of a picture that may be uploaded to the network is 299 by 299 pixels in InceptionV3, whereas in darknet53, the maximum size of a picture that may be uploaded to the network is 256 by 256 pixels. We retrained both of these networks on the HAM10000 dataset using transfer learning in order to obtain more relevant features.

To extract features from the model, we first had to retrain the models using the augmented dataset. For finetuning we first removed the last three layers of both models and added a new layer for number of classes. Transfer learning was used for retraining (TL). Transfer learning (TL) is a method that involves a pre-trained model that is used again for a different classification task [18]. TL has been known to perform well on a variety of classification tasks [19,20,21,22]. Transfer learning updates the weights of the target models, which in our case are InceptionV3 and darknet53, and the dataset used was HAM10000. After reusing both models, two new models for the categorization of multiclass skin lesions were created. During the TL phase, we chose 50% of the photos for training the model and the other 50% for testing. Transfer learning involves the following steps:

- From among the available models, a pre-trained source model is selected for use. Many research institutes publish models based on large datasets. These models could be added to the pool of candidate models from which to choose one.
- After the model has been pre-trained, it may be utilised as the basis for another model that will perform the second job of interest. Depending on the modelling approach that was used, this might require using the entire model or only a portion of it.
- There is also a chance that the model will need to be changed or made better based on the easily accessible input–output data for the activity of interest.

### 3.4. Feature Extraction Using CNN’s

After retraining, the next step was to extract features from both models. Features are extracted from the dataset by applying activations to the deep layers of both models. In CNN models, when we apply activations on the first convolution layer, we obtain features such as colour and edges. In order to extract deep features, we apply activations to deeper layers of models. The deeper layer contains features built up from previous layers.

After retraining the models, we extracted the features by applying activations to “Global Average Pooling”, as it contains features from all previous layers and helps us in extracting the more complex and deep features. We extracted 1024 features for each image in the test and training set using darknet53. We extracted 2048 features for each image in the test and training set using Inception V3.

To make a fair comparison with the existing approach, we performed an additional experiment in which we used the nasnet-large model proposed in the state-of-the-art architecture, retrained it on our dataset using transfer learning, and then extracted statistical features from images using the retrained model. We utilised the characteristics extracted by activating the “Average Global Pooling Layer” to perform classification. To compare the outcomes with our technique, we also decreased the features of Nasnet-large, utilising moth flame optimization.

### 3.5. Moth-Flame Optimization

Moth flame is a population-based algorithm. It was proposed in 2016. In MFO, we assume that the flames are candidate solutions and moths are the problem variables in the space. Moths fly in one, two, or hyper-dimensional space, changing their position vectors. We used MFO in our research to reduce the number of features by selecting the best optimum features from the feature set. Table 1 shows the number of features reduced after using moth flame optimization.

(1) $$cost=alpha\cdot error+beta\cdot (num_{feat}/max_{feat})$$

Equation (1) shows the cost function for the cost calculation of moth flame. The cost function is used to find the optimal value by calculating the global minima. In Equation (1), “*alpha*” and “*beta*” are hyper-parameters. “$num_{feat}$” and “$max_{feat}$” are the total number of features selected by the algorithm and the total number of features passed to the MFO. The error can be calculated using Equation (2).

(2) error=1−Acc

(3) Acc=sum(pred==yvalid)/length(yvalid)

## 4. Experiments and Results

### 4.1. Experimental Setup

In this part, we present the procedures and parameters used to compute the findings. During the testing procedure, we trained the model using 50% of the photos, which were ground truth photos made publicly accessible for research reasons. During the classification phase, we used 50% images for training and the other 50% for testing. The testing results are computed based on K-fold cross validations where the value of K is 10. Several classifiers were used throughout the validation phase. We used a learning rate of 0.001 and a mini-batch size of 8 for the learning procedure. All simulations of the proposed framework were carried out on a desktop computer equipped with 16 GB of RAM and a 256 GB SSD. As a simulation tool, MATLAB R2020a was employed. The desktop computer used was core i5 with 8 cores, and due to the lack of a GPU, it took 15 days to completely train both models for feature extraction.

### 4.2. Results

The results for classification were calculated based on the extracted features from the CNN model that was utilised, and this was performed after feature reduction. The performance of a number of different classifiers was evaluated and compared. These classifiers comprised linear, cubic, and quadratic SVMs; a linear discriminant; fine, medium, and coarse K-Nearest Neighbor; ensemble subspace discriminant; and ensemble subspace KNN. To figure out how well it worked, the time it took to classify both the full set of features and the reduced set was also noted.

Results demonstrated in Table 2 were calculated for three different experiments.

- Classification on the original dataset that had 10,015 images.
- Classification on the augmented dataset that had 12,981 images.
- Lastly, for a fair comparison, we reproduced the results of previously performed work on our dataset.

#### 4.2.1. Results on Original Dataset

In Table 2, results from before the augmentation are demonstrated after applying the moth flame optimization. From these experiments, we achieved the highest accuracy of 90.5% on quadratic SVMs using the merged feature vectors from both CNN models, inception and darknet53, which compared to previous work was less in both accuracy and efficiency. The low accuracy was due to a highly imbalanced dataset. The uneven distribution of images among various classes increased the chance of incorrect classification. This forced us to come up with a solution that may help in improving the accuracy and efficiency of our technique. After further study, we found that image augmentation and balancing the dataset helps in improving the accuracy.

#### 4.2.2. Results on Augmented Data

**Figure 3:**
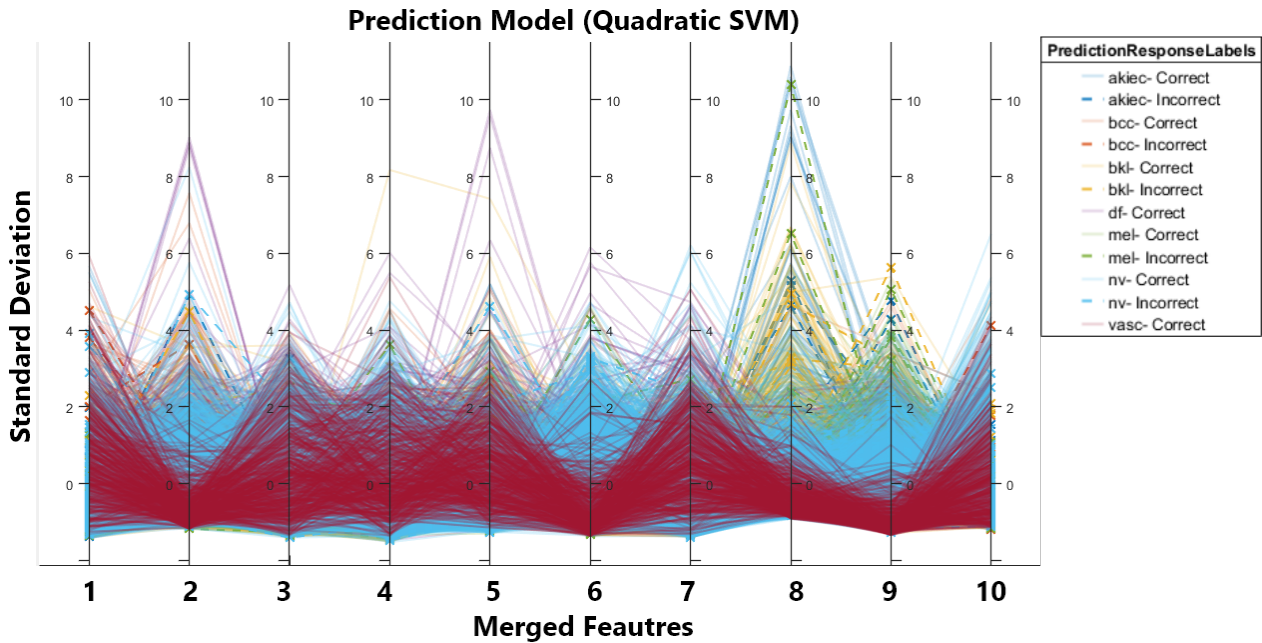
Parallel Co-ordinates Graph.

In order to improve the results, we conducted the second experiment in which we first balanced the dataset using augmentation. We applied three different types of transformations on the dataset, i.e., brightness, contrast enhancement, and sharpening. After augmentation, we had a more balanced dataset with a total of 12,981 images. After that, the augmented dataset was fed into two CNNs, inceptionV3 and darknet53, for feature extraction and classification. Table 2 first displays the classification results for individual optimised feature vectors obtained from CNNs. Secondly, it shows the results for the merged feature vector without optimization and, third, for the optimized and merged feature vectors. It can be seen that there is prominent improvement in accuracy and efficiency. The accuracy of inceptionV3 increased from 90% to 92.7% due to increased training data. Similarly, the same trend is observed for darknet53, i.e., an increase in accuracy and improved efficiency. We achieved 950.9% accuracy on quadratic SVM by merging both feature vectors, and to improve the efficiency of merged vectors, we implemented MFO and again calculated the classification, which resulted in a 0.1% drop in accuracy. So, we achieve 95.8% accuracy for the optimised feature vector on SVM with time reduced to 1/3. The drop in accuracy is due to MFO as we are selecting optimal features that end in removing irrelevant features (reducing the number of features) and causing a minor drop in accuracy. Figure 3 shows the parallel coordinate graph generated for multivariate data in our case feature set. The solid and dashed lines in the graph show correct and incorrect classifications for different classes, and solid lines are used to represent correct classifications, whereas dashed lines are used to represent incorrect classifications.

**Figure 4:**
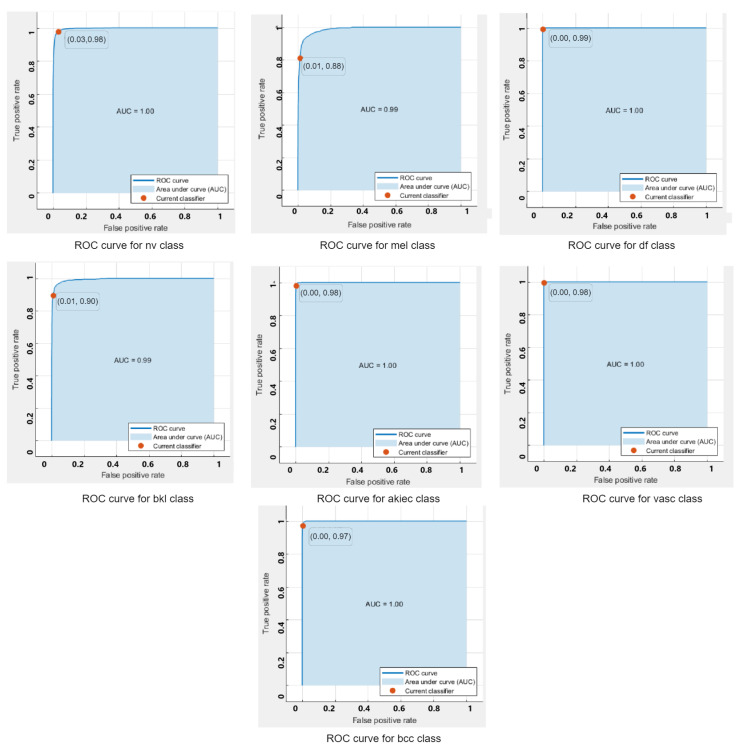
ROC -Curves for each class on SVM.

ROC curves are used to show the performance of a classifier. The ROC-curves of the quadratic SVM for each class classified using the feature extracted from the augmented dataset are shown in Figure 4. It can be seen that all classifers are lying on the top left side of the curves, which indicates a better performance of the model for the clasification of multiple classes.

#### 4.2.3. Results of Existing Approach

Another researcher Khan et al. [14] conducted experiments that involved segmentation and saliency calculations to improve the results of multi-class classification. They achieved 90.67%, and the results are shown in Table 3. From the table, it can be observed that our technique outperformed previously existing approaches that use the segmentation of lesions.

#### 4.2.4. Classification Using Nasnet-large

The last experiment that we conducted aimed to compare the existing approach with our technique. In order to carry that out, we trained the nasnet-large model on our dataset with the same parameters. Table 2 shows the results obtained using nasnet-large under the same parameters and on the same dataset that we used. It achieved 85.5% accuracy on quadratic SVM. Our technique not only outperformed Nasnet-large in terms of accuracy but also in efficiency.

## 5. Discussion

After completing the experimentation, we gathered the following insight from Section 5. Firstly, we can clearly see that augmentation helps in improving the performance of a model used for classification as it increases the amount of data for the model to learn from. In our case, we used augmentation to balance the classes and increase the accuracy and efficiency. From Table 2, it can be seen that accuracy achieved after using the features extracted from the augmented dataset is 5% more accurate than that achieved before augmentation on the original HAM10000 dataset. Secondly, upon comparing the results of our approach with present research studies that use nasnet large for feature extraction, when trained on our augmented dataset, those approaches achieved 85.5% accuracy in 370.72 s, whereas in our approach, we achieved 95.8% in 153.96 s. Thirdly, when we compare the results of our technique with the results of existing techniques Table 3, we can see that our approach outperformed it by almost 5% in term of accuracy. From this, it can be seen that using hybrid features extracted from two different CNNs helps in improving the accuracy of multi-class classification.

## 6. Conclusions and Future Work

In this paper, we proposed an automated approach for the classification of multiple classes of skin lesions based on augmentation as well as CNN feature extraction and feature reduction. The proposed method was evaluated on the HAM10000 dataset, and after comparing it to existing methods, we discovered that it outperformed existing state-of-the-art approaches in terms of both accuracy and efficiency. In our findings, data augmentation helped in improving the accuracy of CNN models. In order to perform classification, the models that were trained on augmented data were used to extract features from InceptionV3 and Darknet53, respectively. The moth flame optimization, which assisted in lowering the number of features while simultaneously enhancing efficiencies, achieved 95.9%, 95%, and 95.8% on cubic SVM, quadratic SVM, and the ensemble subspace discriminant, respectively.

In future, we aim to improve the quality of our work by proposing other possible methods for the segmentation of lesions. In this way, the lesion can be recognised from the background more easily. This would help get rid of irrelevant features that are recognised in the images, which would improve the accuracy and efficiency of the classification. 

## Figures and Tables

**Table 1:** MFO Results of Reduced Features.

Model	Original Features	Reduced Features
**Inception V3**	2048	1031
**Darknet53**	1024	535

**Table 2:** Classification Results.

Classification Model			Quadratic SVM	Cubic SVM	Ensemble Subspace Discriminant	Linear Discriminant	Fine KNN
**Before Augmentation**	**Reduced Merged Feature (MFO)**	**ACC (%)**	**90.5**	**90.3**	**90.4**	88.5	77.8
		**RE (%)**	**83.51**	**72.87**	**78.38**	75.09	60.54
		**PR (%)**	**87.21**	**87.15**	**74.75**	82.7	57.65
		**Time (s)**	**188.8**	**214.2**	**296.5**	76.8	92.64
**After Augmentation**	**InceptionV3**	**ACC (%)**	**92.7**	**92.3**	**90.7**	90.4	88.5
		**RE (%)**	**90.28**	**90.62**	**87.4**	87.34	86.51
		**PR (%)**	**91.61**	**92.01**	**88.27**	88.1	85.2
		**Time (s)**	**154.14**	**164.14**	**491.29**	86.14	84.29
	**Darknet53**	**ACC (%)**	**90.4**	**90.3**	**90.7**	87.9	77.6
		**RE (%)**	**73.04**	**71.25**	**77.48**	74.4	61.74
		**PR (%)**	**86.37**	**86.75**	**85.21**	72.08	59.01
		**Time (s)**	**143.37**	**184.7**	**339.5**	47.4	59.29
	**Without MFO**	**ACC (%)**	**95.9**	**95.9**	**95.8**	92.0	86.2
		**RE (%)**	**94.65**	**94.57**	**93.48**	91.67	86.48
		**PR (%)**	**95.11**	**95.11**	**95.1**	90.6	82.68
		**Time (s)**	**496.02**	**541.1**	**1524.0**	289.36	412.34
	**With MFO**	**ACC (%)**	**95.8**	**95.7**	**95.5**	90.4	86.3
		**RE (%)**	**91.75**	**92.05**	**88.27**	88.12	84.47
		**PR (%)**	**89.97**	**90.5**	**87.42**	87.3	86.02
		**Time (s)**	**153.96**	**166.29**	**667.54**	164.59	100.93
**Nasnet-Large**	**Reduced Features (MFO)**	**ACC (%)**	**85.5**	**79.34**	**77.65**	74.41	75.9
		**RE (%)**	**79.14**	**85.6**	**83.3**	80.8	80.3
		**PR (%)**	**82.04**	**82.3**	**788.22**	75.5	73.45
		**Time (s)**	**370.72**	**435.02**	**671.88**	107.89	199.6

**Table 3:** Results of existing approaches on the HAM1000 dataset.

Classifier	Accuracy (%)	Time (s)
Naive Bayes	81.34	153.30
ELM	84.92	138.50
**KELM**	**90.67**	**133.44**
MSVM	85.50	121.52
Fine KNN	82.08	139.38

## Data Availability

Due to sensitivity of data, we are unable to provide the data.

## Abbreviations

The following abbreviations are used in this manuscript:

CNN	Convolutional neural network;
MFO	Moth flame optimization;
FS	Feature selection;
SVM	Support vector machine;
KNN	K-nearest neighbour;
CAD	Classification and detection;
LCcHIV	Local color-controlled histogram intensity values;
HAM10000	Human against machine with 10,000 training images.
